# Prevalence and Associated Factors of Portopulmonary Hypertension in Patients with Portal Hypertension: A Case-Control Study

**DOI:** 10.1155/2021/5595614

**Published:** 2021-04-20

**Authors:** Yueming Shao, Xin Yin, Tingting Qin, Ruihua Zhang, Yu Zhang, Xiaoyu Wen

**Affiliations:** ^1^Department of Hepatology, First Hospital of Jilin University, Xinmin Street, No. 71, Changchun, Jilin Province 130021, China; ^2^Center for Infectious Diseases, West China Hospital of Sichuan University, Chengdu, Sichuan Province 610041, China

## Abstract

**Background and Aims:**

There are few studies on the prevalence and clinical characteristics of portopulmonary hypertension (POPH) in patients with portal hypertension. In addition, invasive right heart catheterization further limits the clinical diagnosis of POPH patients.

**Methods:**

From January 2018 to December 2019, 1004 patients with portal hypertension were treated in the Department of Hepatology, the First Hospital of Jilin University. Based on the inclusion and exclusion criteria, 188 patients with portal hypertension were finally included. We collected complete clinical data, laboratory examinations, and imaging examinations. Patients were divided into a POPH group and a non-POPH group based on echocardiographic results. We calculated the prevalence of POPH in patients with portal hypertension. The differences in clinical characteristics of the two groups of patients were compared.

**Results:**

The prevalence of POPH in patients with portal hypertension was 2.8%. Among the 188 patients with portal hypertension with fingertip oxygen saturation < 95% at rest, 28 patients had POPH (12 males and 16 females), with an average age of 63 ± 8, and 160 patients did not have POPH (110 males, 50 women), with an average age of 59 ± 11. The proportion of women in the POPH group (*P* < 0.01) and patients without liver cancer (*P* = 0.044) was high. Compared to patients without POPH, patients with POPH had lower hemoglobin (related to the severity of anemia, *P* < 0.01), higher creatinine (*P* < 0.05), and lower partial pressure of oxygen and carbon dioxide (*P* < 0.05). Patients with POPH had a higher incidence of atrial enlargement, ventricular enlargement, mitral valve regurgitation, tricuspid regurgitation, pulmonary artery widening, pericardial effusion, and aortic regurgitation than those without POPH. The risk of POPH did not increase with the aggravation of the Child-Pugh classification.

**Conclusion:**

The prevalence of POPH in patients with portal hypertension is 2.8%. The proportion of women and nonliver cancer in POPH patients was higher than that in non-POPH patients. In addition, the POPH group had higher creatinine and lower hemoglobin, and echocardiography showed that POPH patients had more cardiac structural changes. In patients with portal hypertension, the risk in patients with POPH has nothing to do with the Child-Pugh classification and MELD score.

## 1. Introduction

Portopulmonary hypertension is defined as pulmonary hypertension formed on the basis of portal hypertension, which is common in liver cirrhosis, bile duct obstruction, and cholestatic diseases. The diagnosis of POPH relies on right heart catheterization. According to the latest guidelines, mean pulmonary artery pressure (mPAP) is higher than 20 mmHg (1 mmHg = 0.133 KPa), pulmonary vascular resistance (PVR) is higher than 240 dyn s cm^−5^ (=3 Wood units), and pulmonary capillary wedge pressure (PCWP) is less than 15 mmHg [1].

Due to the low incidence of the disease, the current understanding of the disease remains unclear. On account of the limited clinical data, descriptions of the clinical features of the disease are rare and inconsistent. The prevalence of POPH in patients with portal hypertension ranges from approximately 1% to 2% [2], and the prevalence of POPH in liver transplant patients increases from 5% to 8% [3]. The pathogenesis of POPH is not yet clear. At present, the mechanism generally acknowledged by scholars is the imbalance of vasoactive substances [4–6]. Diastolic vasoactive substances decreased, while systolic vasoactive substances increased, resulting in the balance being broken. The clinical manifestations of POPH are nonspecific. The main manifestation is different degrees of dyspnoea. One-third of patients may experience transient syncope. Owing to the invasion of right heart catheterization, echocardiography is often used clinically as a screening tool, and its sensitivity and specificity are shown to be high [7]. The treatment of POPH is mainly targeted therapy of pulmonary hypertension and liver transplantation. The 5-year survival rate of untreated POPH patients is 14.2% [8]. At present, the prognosis of POPH has been improved after liver transplantation and targeted therapy of pulmonary hypertension. The incidence of POPH is relatively low, and there are currently few reports on the clinical features and risk factor analysis of the disease in China. One previous study performed in China was focused on POPH in liver transplant recipients [8]. We conducted two consecutive years for patients with portal hypertension, whose study population is broader. Screening was performed to further clarify the incidence and clinical characteristics of POPH. We hope to provide clinical guidance for the early diagnosis of POPH through this research and to improve clinicians' understanding of POPH.

## 2. Materials and Methods

### 2.1. Study Population

**Figure 1:**
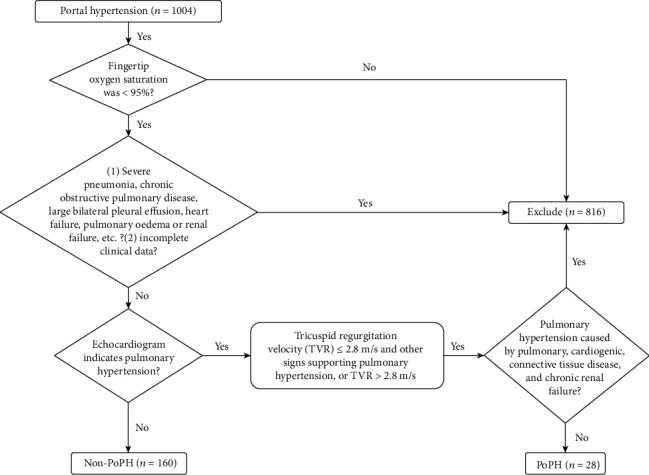
Study screening flowchart.

From January 2018 to December 2019, 188 patients with portal hypertension (selected from 1004 patients with portal hypertension) who were admitted to the Department of Hepatology, the First Hospital of Jilin University, whose fingertip oxygen saturation was less than 95%, were enrolled in this research (Figure 1). The exclusion criteria were as follows: (1) patients with nonportal hypertension; (2) patients with severe pneumonia, chronic obstructive pulmonary disease, large bilateral pleural effusion, heart failure, pulmonary oedema, renal failure, etc., whose fingertip blood oxygen saturation was less than 95% in the resting state; (3) patients with pulmonary hypertension caused by pulmonary, cardiogenic, and connective tissue disease and chronic renal failure; and (4) patients with incomplete clinical data. The research protocol of this study was approved by the Ethics Committee of the First Hospital of Jilin University and was conducted in accordance with the Declaration of Helsinki (No. 2020-592). Informed consent was waived by the Institutional Review Board due to the retrospective nature of the study with no more than minimal risk.

### 2.2. Data Collection

We collected general information (sex, age, etiology, etc.) of the above patients, laboratory tests (complete blood count, coagulation function, liver function, renal function, etc.), and auxiliary examinations (echocardiography, abdominal computed tomography (CT) examination, chest CT, etc.). Echocardiography was performed according to the recommendations of the American Society of Echocardiography in 2015, and the patients were divided into the POPH group and the non-POPH group according to the 2015 ESC/ERS pulmonary hypertension diagnosis and treatment guidelines. We compared the differences in clinical characteristics, laboratory tests, and echocardiography between the two groups.

### 2.3. Diagnosis of POPH

The diagnosis of POPH is based on the 2015 ESC/ERS pulmonary hypertension guidelines. Patients with echocardiography indicating medium-to-high risk (tricuspid regurgitation velocity ≤ 2.8 m/s and other signs supporting pulmonary hypertension or tricuspid regurgitation velocity > 2.8 m/s) and excluding other causes of pulmonary hypertension were included in the POPH group, and the remaining patients were included in the non-POPH group.

### 2.4. Statistical Analysis

All data were statistically analyzed using SPSS 22.0 software (IBM ® Corporation, USA). Measurement data conforming to a normal distribution were described by the mean ± standard deviation. Comparisons between the two groups were performed by *t*-tests. Data not conforming to a normal distribution were taken as the median. The number and interquartile range P50 (P25-P75) were also used for description. The Mann-Whitney *U* test was used between these two groups. The enumeration data and grade data were described by the composition ratio, and the chi-square test was used for the comparison of the enumeration data between the two groups. The chi-square test was used to compare the grade data between the two groups. The correlation of grade data was analyzed by the trend chi-square test, and *P* < 0.05 indicated statistical significance.

## 3. Results and Discussion

### 3.1. Clinical Features

In this study, a total of 1004 patients with portal hypertension were screened, and 188 patients with portal hypertension with fingertip oxygen saturation less than 95% were included. Twenty-eight of these were diagnosed with POPH. The patients were not targeted for pulmonary hypertension treatment before and after diagnosis. The proportion of female patients and patients without liver cancer in the POPH group was higher than that in the non-POPH group, and there was no significant difference in other aspects (Table 1). Data are provided as number of cases (%) (HBV: hepatitis B virus, HCV: hepatitis C virus).

### 3.2. Laboratory Examination

A statistical analysis of the laboratory test results of the two groups showed that the proportion of patients with anemia in the POPH group was higher than that of patients without POPH. The two groups also had differences in the distribution of anemia, and the mean corpuscular hemoglobin (MCH) and mean corpuscular hemoglobin concentration (MCHC) in the POPH group were lower. However, the mean corpuscular volume (MCV) of the non-POPH group was not significantly different from that of the POPH group. In addition, the POPH group had lower partial pressure of oxygen (PaO_2_), partial pressure of carbon dioxide (PaCO_2_), and potassium ions and higher creatinine (CREA) values than patients without POPH (Table 2). Data are provided as the median (IQR) and number of cases (%) (ALT: alanine aminotransferase, AST: aspartate aminotransferase, ALB: albumin, TBiL: total bilirubin, PT: prothrombin time, CREA: creatinine, PaO_2_: partial pressure of oxygen, PaCO_2_: partial pressure of carbon dioxide, PLT: platelet, HGB: hemoglobin, MCV: mean corpuscular volume, MCH: mean corpuscular hemoglobin, MCHC: mean corpuscular hemoglobin concentration, MELD: model for end-stage liver disease; normal ranges: AST 15-40 U/L; ALT 9-50 U/L; ALP: 45-135 U/L; ALB: 40-55 g/L; TBiL: 0-26.0 *μ*mol/L; PT 9-13 s; CREA: 41-73 *μ*mol/L; PaO_2_: 83-108 mmHg; PaCO_2_: 35-48 mmHg; potassium ion: 3.5-4.5 mmol/L; sodium ion: 136-145 mmol/L; PLT: 125-350 × 10^9^/L; HBG: 115-150 g/L; MCV: 82-100 fL; MCH: 27-34 pg; MCHC: 316-354 g/L).

Calculation formulas are as follows: MELD = 3.8 × ln(bilirubin mg/dl) + 11.2 × ln (INR) + 9.6 × ln(creatinine mg/dl) + 6.4 × (etiology : 0 if cholestatic or alcoholic, 1 otherwise) [10].

### 3.3. Echocardiography

The echocardiography results between the POPH group and the non-POPH group are shown in Table 3. Compared to the non-POPH group, patients in the POPH group showed enlarged atrial and ventricular echocardiography, as well as mitral, tricuspid, and aortic regurgitation, pulmonary artery widening, and pericardial effusion (*P* < 0.05). There were no significant differences between the two groups in terms of pulmonary regurgitation, severity of diastolic function, and systolic function (left ventricular short-axis contraction rate and ejection fraction) (*P* > 0.05).

### 3.4. Correlation between the Risk of Portopulmonary Hypertension and the Child-Pugh Classification and MELD Score

Among 188 patients whose fingertip blood oxygen saturation was less than 95% at rest, 28 cases of POPH were screened. Among them, 16 were intermediate-risk patients, and 12 were high-risk patients. In other words, 4 were Child-Pugh A patients, 14 were Child-Pugh B patients, and 10 were Child-Pugh C patients. The above data were analyzed by the chi-square test trend (Table 4), and it was found that the risk of POPH did not increase with the aggravation of the Child-Pugh classification (*P* = 0.382). The MELD score of patients in the intermedium-risk group was 14.3 ± 8.0, while the MELD score in the high-risk group was 12.4 ± 7.1. The difference in MELD scores between the two groups could not be considered statistically significant (*t* = 0.655, *P* = 0.518) (Table 5).

## 4. Discussion

With the continuous development of current medical technology, the diagnosis of POPH has entered the field of a wide range of clinicians. However, the pathogenesis of POPH is not yet fully understood, and further study and exploration are needed. POPH is more commonly seen in patients with decompensated liver cirrhosis. How to effectively screen for POPH among the huge population of liver cirrhosis patients in China plays a particularly important role in their prognosis. Therefore, we hope to screen key patients through this study to further deepen people's understanding of the disease.

The study included 1004 patients, of which 188 subjects were the main study subjects. The prevalence of POPH among people with portal hypertension who did not undergo liver transplantation was 2.8%. There is no clear report of the incidence in patients with liver cirrhosis, and in patients with advanced liver cirrhosis, the incidence can be as high as 8.5% [11]. Although our research proves that there is no relationship between the risk of POPH and the Child-Pugh classification and MELD score of patients, studies have shown that the correlation between POPH and the severity of liver disease is weak [12]. The mechanism of POPH risk in patients with advanced liver disease may be related to genetic polymorphisms of oestrogen-related genes and other pathways involving cell growth/apoptosis and oxidative stress [13]. The POPH group was more common in female patients, which may be related to the involvement of oestrogen in the disease [14]. Previous studies have proven that woman can be a risk factor for POPH [15].

Anemia is more common in patients in the POPH group. This may be because patients with chronic anemia require higher resting cardiac output to compensate for the decreased oxygen-carrying capacity, so pulmonary hypertension may also occur in hemolytic diseases [16]. A study is consistent with our results, showing that anemia is an independent risk factor for POPH [17]. Patients with liver cirrhosis and POPH show more valve regurgitation, enlargement of the atria and ventricles, and widening of the pulmonary artery on echocardiography. Therefore, when using echocardiography for POPH screening, attention should be paid to the abovementioned positive results of patients, and the screening and follow-up of the disease should be strengthened.

In patients with portohypertension whose fingertip oxygen saturation is less than 95% in the resting state, the partial pressure of oxygen and carbon dioxide in the POPH group is significantly lower than that in the non-POPH group, which is related to the increased alveolar-arterial oxygen gradient. In addition, the creatinine value and number of nonliver cancer patients in the POPH group were higher than those in the non-POPH group. The correlation between creatinine and liver cancer history and POPH has not been reported, but the increase in creatinine caused by increased visceral circulation load in POPH patients is not excluded, and a large sample is needed for further confirmation.

Our study found that there were no differences in liver function, Child-Pugh classification, MELD score, or etiology between the POPH group and the non-POPH group. This is consistent with the results of a previous study that the Child-Pugh classification is not related to POPH [18]. Studies have shown that increased MELD scores in POPH patients before transplantation are associated with poor survival after transplantation [19], and we need to confirm this conclusion with long-term follow-up observation. Foreign studies have reported that hepatitis C is a protective factor for POPH, but we did not find this in our research. Considering the differences in etiology between China and Western countries, the main cause of the population in China is viral hepatitis B. The main cause of the disease in the country is hepatitis C and alcoholic hepatitis, so the results between the two are not the same [20]. Patients with POPH often have hypoxia. To reduce the economic burden of hospitalized patients, we used fingertip blood oxygen saturation < 95% as the screening index in the resting state. Because a relevant research reported screened population whose arterial blood oxygen pressure was less than 60 mmHg, the sensitivity and specificity of fingertip blood oxygen saturation < 95% in the resting state can be as high as 100% and 93% [21]. The limitations of this study include the following points: (1) the sample size of samples is insufficient, so the number of positive patients is small, and the conclusions drawn need to be confirmed by further expanding the sample size, and (2) the diagnostic standard used in this study is echocardiography. Due to various reasons, right heart catheterization could not be performed for further confirmation.

In summary, to further clarify the clinical characteristics and predisposing factors of POPH patients, we still need to increase the sample size to analyze whether there are real differences in clinical characteristics between the two populations, and the reasons for the differences need to be further explored. In addition, in future research, we will further expand the sample size, explore the correlation between POPH and portal hypertension parameters, and deepen our understanding of POPH.

## 5. Conclusions

Our study provides estimates of the prevalence of POPH in patients with portal hypertension. The proportion of women with nonliver cancer in POPH patients is higher than that in non-POPH patients. The POPH group had higher creatinine and lower hemoglobin. Echocardiography showed greater changes in the heart structure of POPH patients.

## Figures and Tables

**Table 1:** Analysis of demographic situation of the POPH group and non-POPH group.

Variables	POPH (*n* = 28)	Non-POPH (*n* = 160)	*χ* ^2^/*t*	*P*
Age (years)	63 ± 8	59 ± 11	-1.541	0.125
Gender *N* (%)				
Male	12 (42.9)	110 (68.7)	7.017	<0.01
Female	16 (57.1)	50 (31.3)		
Etiology *N* (%)				
HBV	10 (35.7)	71 (44.4)	0.729	0.393
HCV	2 (7.1)	21 (13.1)	—	0.537
Alcohol	9 (32.1)	37 (23.1)	1.049	0.306
Drug	1 (3.6)	6 (3.8)	—	1.00
Autoimmune	3 (10.7)	14 (8.8)	—	0.723
Liver cancer *N* (%)				
Yes	4 (14.3)	51 (31.9)	3.562	0.059
No	24 (85.7)	109 (68.1)		
Hypertension *N* (%)				
Yes	5 (17.9)	22 (13.7)	—	0.563
No	23 (82.1)	138 (86.3)		
Diabetes *N* (%)				
Yes	3 (10.7)	30 (18.8)	—	0.422
No	25 (89.3)	130 (81.3)		
Splenectomy *N* (%)				
Yes	6 (21.4)	21 (13.1)	—	0. 249
No	22 (78.6)	139 (86.9)		
Portal vein thrombosis *N* (%)				
Yes	4 (14.3)	21 (13.1)	—	0.771
No	24 (85.7)	139 (86.9)		

The diagnosis of portal hypertension in this study is based on the 2016 Practice Guidance by the AASLD on Portal Hypertensive Bleeding in Cirrhosis [9]. All patients had manifestations of liver cirrhosis and portal hypertension confirmed by abdominal imaging (ultrasound, CT, or MRI), and most patients had complications of portal hypertension at the time of admission, such as portal hypertensive bleeding and ascites.

**Table 2:** Analysis of laboratory examination of the POPH group and non-POPH group.

Variables	POPH (*n* = 28)	Non-POPH (*n* = 160)	*z*/*t*	*P*
ALT (U/L)	21.8 (14.3-21.8)	33.9 (19.6-59.9)	-1.451	0.147
AST (U/L)	43.9 (30.9-64.7)	53.7 (34.2-91.9)	-1.043	0.297
ALB (g/L)	27.6 ± 5.7	28.4 ± 5.9	0.701	0.484
TBiL (*μ*mol/L)	34.8 (18.5-95.4)	40.7 (24.1-101.6)	-1.065	0.287
PT (s)	15.5 (13.6-19.3)	15.0 (13.2-18.9)	-0.776	0.438
CREA (mmol/l)	80.0 (59.0-99.0)	65.1 (52.3-80.0)	-2.091	0.037
PaO_2_ (mmHg)	67.3 (58.0-77.9)	72.7 (65.4-81.8)	-2.166	0.030
PaCO_2_ (mmHg)	31.0 (27.7-32.9)	33.0 (31.0-36.0)	-2.524	0.012
Potassium ion (mmol/l)	3.6 (3.2-3.8)	3.9 (3.5-4.2)	-2.810	<0.01
Sodium ion (mmol/l)	136.1 (133.6-138.9)	135.9 (132.4-139.0)	-0.631	0.528
PLT (×10^9^/L)	84.0 (51.0-121.0)	86.0 (60.0-127.0)	-0.346	0.729
HGB (g/L)	95.2 ± 25.5	112.3 ± 31.0	2.744	<0.01
MCV (fl)	93.0 (86.3-98.1)	96.1 (90.6-101.8)	-1.771	0.077
MCH (pg)	31.4 (27.8-33.5)	32.6 (30.7-35.5)	-2.235	0.025
MCHC (g/L)	330.0 (315.3-343.5)	341.0 (329.3-350.0)	-2.457	0.014
The degree of anemia *N* (%)				
Normal	5 (17.8)	79 (49.4)	-2.740	<0.01
Mild anemia	11 (39.3)	39 (24.4)		
Moderate anemia	11 (39.3)	34 (21.3)		
Severe anemia	1 (3.6)	8 (5.0)		
Child-Pugh classification *N* (%)				
A	4 (14.3)	25 (15.6)	-0.012	0.990
B	14 (50)	76 (47.5)		
C	10 (35.7)	59 (36.9)		
MELD score	12.5 (10.0-18.5)	11.0 (8.0-17.0)	-0.932	0.351

**Table 3:** Analysis of laboratory examination of the POPH group and non-POPH group.

Echocardiography	POPH (*n* = 28)	Non-POPH (*n* = 160)	*χ* ^2^/*z*	*P*
Enlargement of the left atrium *N* (%)				
Normal	10 (35.7)	132 (82.5)	28.224	<0.01
Enlargement	18 (64.3)	28 (17.5)		
Enlargement of the left ventricle *N* (%)				
Normal	22 (78.6)	153 (95.6)	—	<0.01^∗^
Enlargement	6 (21.4)	7 (4.4)		
Enlargement of the right atrium *N* (%)				
Normal	13 (46.4)	165 (96.9)	—	<0.01^∗^
Enlargement	15 (53.6)	5 (3.1)		
Enlargement of the right ventricle *N* (%)				
Normal	25 (89.3)	158 (98.8)	—	0.024^∗^
Enlargement	3 (10.7)	2 (1.2)		
Pericardial effusion *N* (%)				
Yes	12 (42.9)	12 (7.5)	—	<0.01^∗^
No	16 (57.1)	148 (92.5)		
Mitral regurgitation *N* (%)				
Yes	16 (57.1)	25 (15.6)	24.088	<0.01
No	12 (42.9)	135 (84.4)		
Aortic regurgitation *N* (%)				
Yes	8 (28.6)	17 (10.6)	—	0.016^∗^
No	20 (71.4)	143 (89.4)		
Pulmonary regurgitation *N* (%)				
Yes	1 (3.6)	1 (0.6)	—	0.276^∗^
No	27 (96.4)	159 (99.4)		
Tricuspid regurgitation *N* (%)				
Yes	26 (92.9)	37 (23.1)	52.006	<0.01
No	2 (96.4)	123 (76.9)		
Pulmonary artery widening *N* (%)				
Yes	10 (35.7)	0 (0)	—	<0.01^∗^
No	18 (64.3)	160 (100.0)		
Diastolic function *N* (%)				
Normal	2 (7.1)	28 (17.5)	-1.781	0.075
Suspicious reduction	6 (21.4)	45 (28.2)		
Reduction	20 (57.5)	87 (54.3)		
Left ventricular short-axis contraction rate (%)	32 (31-33)	32 (30-33)	-1.008	0.313
Ejection fraction (%)	60 (59-64)	60 (60-63)	-0.653	0.514

^∗^Fisher's exact test.

**Table 4:** Correlation between the risk of portal hypertension and Child-Pugh classification.

Risk level	Child-Pugh A (*n* = 4)	Child-Pugh B (*n* = 14)	Child-Pugh C (*n* = 10)	*P*
Medium	2	7	7	0.382
High	2	7	3	

**Table 5:** Correlation between the risk of portal hypertension and MELD score.

Risk level	Medium (*n* = 16)	High (*n* = 12)	*P*
MELD score	14.3 ± 8.0	12.4 ± 7.1	0.518

## Data Availability

The datasets used and/or analyzed during the current study are available from the corresponding author upon reasonable request.
